# Dietary weight loss and exercise interventions effects on quality of life in overweight/obese postmenopausal women: a randomized controlled trial

**DOI:** 10.1186/1479-5868-8-118

**Published:** 2011-10-25

**Authors:** Ikuyo Imayama, Catherine M Alfano, Angela Kong, Karen E Foster-Schubert, Carolyn E Bain, Liren Xiao, Catherine Duggan, Ching-Yun Wang, Kristin L Campbell, George L Blackburn, Anne McTiernan

**Affiliations:** 1 Public Health Sciences Division, Fred Hutchison Cancer Research Center, Seattle, WA, USA; 2 Office of Cancer Survivorship, National Cancer Institute, National Institutes of Health, Bethesda, MD, USA; 3 Cancer Education and Career Development Program, University of Illinois at Chicago, Chicago, IL, USA; 4 Department of Medicine, School of Medicine, University of Washington, Seattle, WA, USA; 5 Department of Biostatistics, School of Public Health, University of Washington, Seattle, WA, USA; 6 Department of Physical Therapy, University of British Columbia, Vancouver, BC, Canada; 7 Department of Surgery, Beth Israel Deaconess Medical Center, Harvard Medical School, Boston, MA, USA; 8 Department of Epidemiology, School of Public Health, University of Washington, Seattle, WA, USA

**Keywords:** health-related quality of life, exercise, dietary weight loss, postmenopausal women

## Abstract

**Background:**

Although lifestyle interventions targeting multiple lifestyle behaviors are more effective in preventing unhealthy weight gain and chronic diseases than intervening on a single behavior, few studies have compared individual and combined effects of diet and/or exercise interventions on health-related quality of life (HRQOL). In addition, the mechanisms of how these lifestyle interventions affect HRQOL are unknown. The primary aim of this study was to examine the individual and combined effects of dietary weight loss and/or exercise interventions on HRQOL and psychosocial factors (depression, anxiety, stress, social support). The secondary aim was to investigate predictors of changes in HRQOL.

**Methods:**

This study was a randomized controlled trial. Overweight/obese postmenopausal women were randomly assigned to 12 months of dietary weight loss (n = 118), moderate-to-vigorous aerobic exercise (225 minutes/week, n = 117), combined diet and exercise (n = 117), or control (n = 87). Demographic, health and anthropometric information, aerobic fitness, HRQOL (SF-36), stress (Perceived Stress Scale), depression [Brief Symptom Inventory (BSI)-18], anxiety (BSI-18) and social support (Medical Outcome Study Social Support Survey) were assessed at baseline and 12 months. The 12-month changes in HRQOL and psychosocial factors were compared using analysis of covariance, adjusting for baseline scores. Multiple regression was used to assess predictors of changes in HRQOL.

**Results:**

Twelve-month changes in HRQOL and psychosocial factors differed by intervention group. The combined diet + exercise group improved 4 aspects of HRQOL (physical functioning, role-physical, vitality, and mental health), and stress (p ≤ 0.01 vs. controls). The diet group increased vitality score (p < 0.01 vs. control), while HRQOL did not change differently in the exercise group compared with controls. However, regardless of intervention group, weight loss predicted increased physical functioning, role-physical, vitality, and mental health, while increased aerobic fitness predicted improved physical functioning. Positive changes in depression, stress, and social support were independently associated with increased HRQOL, after adjusting for changes in weight and aerobic fitness.

**Conclusions:**

A combined diet and exercise intervention has positive effects on HRQOL and psychological health, which may be greater than that from exercise or diet alone. Improvements in weight, aerobic fitness and psychosocial factors may mediate intervention effects on HRQOL.

## Background

Nearly two-thirds of US adults are overweight or obese [1]. These individuals are at increased risk for a variety of chronic diseases including metabolic disease, heart disease, cancer, and psychosocial disorders [2], which may significantly reduce health-related quality of life (HRQOL). A review of 8 studies examining HROQL among women aged over 55 years old concluded that postmenopausal women, especially those with BMI greater than 30 kg/m^2^, have lower HRQOL in physical functioning, energy, and vitality compared with normal-weight women [3].

Lifestyle modification including dietary weight loss or physical activity has been shown to improve HRQOL [4-6]. Despite the numbers of studies reporting positive effects of lifestyle modification on HRQOL, limited studies have investigated possible mechanisms of change in HRQOL. Further, the optimal lifestyle prescription for improving HRQOL has not been established [7].

Increasing evidence suggests that the combination of diet and exercise may be superior to diet or exercise alone with respect to reducing weight [8,9], improving lipid profile [10,11] and preventing type 2 diabetes [12]. However, the few intervention studies that compared the effects of dietary weight loss and/or exercise interventions on HRQOL have shown mixed results [13-15]. Among 76 patients with type 2 diabetes, diet+exercise and diet-only intervention groups significantly improved in a general quality of life measure [13]. In 316 older adults with osteoarthritis, individuals assigned to a diet+exercise intervention improved HRQOL (physical functioning, general health, role-physical, body pain, and social functioning) compared with controls [14]. Among 157 healthy men, no differences in change in HRQOL were observed among men randomized to diet+exercise, diet-only, exercise-only, or control groups [15].

Despite numerous exercise and dietary weight loss interventions reporting positive changes in HRQOL, the mechanisms behind how exercise and dietary weight loss programs improve HRQOL are not clear. While some intervention studies have shown that weight loss is associated with improved HRQOL [16,17], others have shown that people improve HRQOL without anthropometric changes [18,19].

The primary aim of this study was to examine the individual and combined effects of dietary weight loss and exercise interventions on HRQOL. Defining the individual and combined effects of diet and exercise interventions on HRQOL will help inform researchers, practitioners and policy makers on optimal lifestyle prescriptions for improving HRQOL. The secondary aim was to explore physical and psychosocial factors associated with changes in HRQOL during the intervention. The findings would provide information to explain potential mechanisms of how diet and exercise interventions affect HRQOL.

## Methods

**Figure 1:**
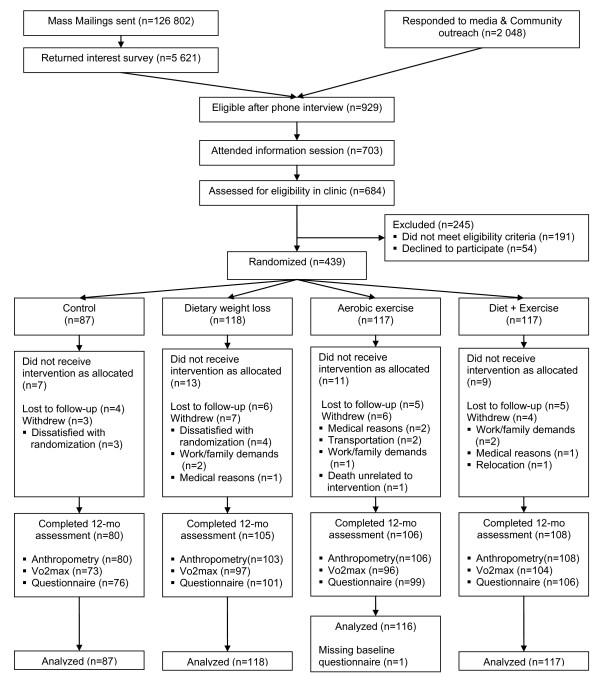
**Flow diagram of the trial**.

The Nutrition and Exercise for Women (NEW) trial was a 12-month, randomized controlled trial conducted at the Fred Hutchinson Cancer Research Center, Seattle, WA from 2005 to 2009. Participants were recruited from the greater Seattle, WA area though mass mailing and media placements from 2005 to 2008, and 439 were enrolled in the study (Figure 1). The study inclusion criteria included: age 50-75 years old; body mass index (BMI) ≥ 25.0 kg/m^2 ^(if Asian-American ≥ 23.0 kg/m^2^); < 100 minutes per week of moderate or vigorous intensity physical activity; postmenopausal; not taking hormone replacement therapy for the past 3 months; no history of breast cancer, heart disease, diabetes mellitus, or other serious medical conditions; fasting glucose < 126 mg/dL; currently not smoking; alcohol intake of fewer than 2 drinks per day; able to attend diet/exercise sessions at the intervention site; and normal exercise tolerance test.

Women were randomized to: (1) dietary weight loss with a goal of 10% weight reduction (N = 118), (2) moderate-to-vigorous intensity aerobic exercise for 45 minutes/day, 5 days/week (N = 117), (3) combined exercise and diet (N = 117), and control groups (N = 87). Study staff performed randomization through a computer program developed by the study statistician. Randomization was blocked on BMI (< 30.0 kg/m^2 ^or ≥ 30.0 kg/m^2^) and race/ethnicity (White, Black, and others). In addition, to achieve a proportionally smaller number of women assigned to the control group, a permuted blocks randomization with blocks of 4 was used, where in the control assignment was randomly eliminated from each block with a probability of approximately 1 in 4. The NEW trial was designed to have sufficient power to detect a difference of 10% change in serum estrone, the primary study outcome, over a 12-month period making three primary pairwise comparisons: diet + exercise vs. exercise; diet + exercise vs. diet; and diet vs. exercise intervention groups. Based on the number of participants who completed the 12-month assessments, we estimate that we have 99.9% power to detect 10 points change in the physical functioning scale (HRQOL).

All study procedures were reviewed and approved by the Fred Hutchinson Cancer Research Center Institutional Review Board in Seattle, WA, and all participants provided signed Informed Consent.

### Interventions

The diet group received a reduced calorie weight loss intervention, a modification of the Diabetes Prevention Program (DPP) lifestyle [20] and Look AHEAD (Action for Health in Diabetes) trial [21] interventions with goals of: total caloric intake of 1200- 2000 kcal/day based on baseline weight, ≤30% calories from fat, and 10% weight loss within the first 24 weeks with maintenance for the rest of intervention period. The diet intervention was conducted by dietitians with training in behavior modification. Participants had individual sessions with the dietitians at least twice, then met weekly in small groups (average 5-10 women) until week 24, and afterward communicated with the dietitians at least twice per month either via group sessions or via email/phone contact. The diet intervention involved sessions designed to develop strategies and skills to achieve caloric and weight loss goals, which included self-monitoring, goal setting, coping strategies, and problem solving.

The exercise intervention was 45 minutes per day of moderate-to-vigorous intensity aerobic exercise, 5 days per week including 3 exercise physiologist-supervised sessions per week at the facility. Over the first 8 weeks, participants gradually increased the intensity and duration of exercise training to 70-85% of maximal heart rate (using Polar heart rate monitors, Lake Success, NY) for 45 minutes per session and maintained this level thereafter.

Women in the diet+exercise group received both the reduced-calorie weight loss and exercise interventions. The diet sessions were provided separately for diet+exercise and diet only groups. Although the diet and exercise group used the exercise facility with women assigned to the exercise-only group, participants were instructed not to discuss the diet intervention.

Controls were not given an intervention during the trial, but were offered 4 group diet sessions and 8 weeks of supervised exercise sessions after 12 months' data collection.

### Measures

Information on demographics, medication use, anthropometrics, aerobic fitness, lifestyle behaviors, psychosocial factors, and HRQOL were assessed at baseline and 12 months. Study staff involved in these assessments were blinded to randomization. Information on age, race/ethnicity, education, marital status, and employment were collected using a standardized questionnaire. Participants were asked to bring their current prescription and over-the-counter medications to the clinic, and information on drug name, dose, frequency, and duration of use were abstracted. Height and weight were measured with a stadiometer and digital scale, and BMI was calculated as kg/m^2^. Aerobic fitness was assessed with a maximum grade treadmill test using the modified branching protocol [22,23]. Physical activity was measured using an interview adapted from the Minnesota Leisure Time Physical Activity Questionnaire [24]. Dietary intake was assessed using the Women's Health Initiative 120-item food frequency questionnaire [25].

Psychosocial factors examined included depression, anxiety, perceived stress, and social support. Depression and anxiety were assessed by the Brief Symptom Inventory-18 [26]. Raw scores were calculated and T scores were assigned according to the scoring manual [27] with higher scores indicating more symptoms of depression and anxiety. Perceived stress was assessed with the Perceived Stress Scale [28]; scores ranged from 0 to 4 with larger scores indicating greater perceived stress. Overall social support was assessed by the short version of the Medical Outcomes Study (MOS) Social Support Survey [6,29]. A mean of all item scores was calculated and converted to a score ranging from 0 to 100. Higher social support scores suggest greater perception of social support. HRQOL was assessed by the MOS 36-Item Short-Form Health Survey (SF-36) [30]. Eight subscales (physical functioning, role-physical, bodily pain, vitality, general health, social functioning, role-emotional, and mental health) were calculated, per standard scoring protocol. Scores ranges from 0 to 100 with higher scores indicating a better state of HRQOL. For the bodily pain subscale, higher scores represent less pain.

### Statistical analyses

We performed analyses using last observation carried forward. For comparison, we also performed the analyses using available data and using multiple imputation. All randomized participants were included in the analyses following the intention-to-treat principle. The baseline characteristics were compared across the 4 study arms using analysis of variance (ANOVA) and chi-square tests, as appropriate. T-tests were used to compare differences in baseline HRQOL and psychosocial factors (depression, anxiety, perceived stress, and social support) by subgroups defined by baseline characteristics: age (defined by median split as < 57 years vs. ≥ 57 years), ethnicity (non-Hispanic White, others), education (no college degree, college degree), employment (employed, unemployed), marital status (no partner, married or with partner), baseline BMI (25 ≤ BMI < 30, ≥ 30 kg/m^2^), and use of antidepressants or anxiolytics (no, yes). Baseline characteristics that significantly altered HRQOL scores and psychosocial factors were included as covariates in the subsequent analyses. We also tested models without these covariates (unadjusted model). The 12-month changes in HRQOL were compared among the 4 study arms using the analysis of covariance (ANCOVA) adjusting for baseline scores and covariates identified in the analysis given above. We used the Bonferroni correction to adjust for multiple comparisons (P-value = 0.05/3 = 0.017 for 3 comparisons).

Data for all participants were used in the following analyses. For HRQOL subscales which significantly differed across intervention groups, Pearson's correlation coefficients were calculated to assess the bivariate associations between changes in HRQOL and physical and psychological factors (weight, aerobic fitness, depression, perceived stress and social support). Multiple regression analysis was used to assess predictors of HRQOL change. All analyses were performed with SAS software (version 9.1; SAS Institute, Cary, NC).

## Results

Baseline questionnaire data was available from 438 participants. Of the 439 women randomized to the 4 study arms, 399 completed physical exams, 370 completed a treadmill test, and 382 returned the questionnaire at 12 months (Figure 1). There were no differences in baseline HRQOL score or psychosocial variables (depression, anxiety, perceived stress, and social support) between those who completed vs. did not complete the 12-months questionnaire (all p-values > 0.05).

### Baseline characteristics of study participants

Table 1 displays the baseline characteristics of the study participants. Participants were a mean age of 58 years; mostly non-Hispanic white (85%); and highly educated (65% with college degree). There were no differences in baseline characteristics among the 4 study arms (all p-values > 0.05). There were no differences in psychosocial factors and HRQL between the four study arms except the mental health score. The exercise group had higher mental health scores compared with diet and control groups at baseline (p < 0.05).

**Table 1:** Baseline characteristics of study participants stratified by trial arm

	Control	Diet	Exercise	Diet+Exercise
	N = 87	N = 118	N = 117	N = 117
**Demographics**				
Age (years), mean (SD)	57.4 (4.4)	58.1 (5.9)	58.1 (5.0)	58.0 (4.5)
Ethnicity, N (%)				
Non-Hispanic white	74 (85.1)	101 (85.6)	98 (83.8)	100 (85.5)
Education, N (%)				
College degree	59 (67.8)	76 (64.4)	70 (59.8)	82 (70.1)
Marital status ^a^, N (%)				
Married or with partner	59 (67.8)	79 (67.0)	71 (60.7)	70 (60.3)
Employment ^b^, N (%)				
Employed	72 (97.3)	92 (87.6)	87 (90.6)	94 (91.2)
Unemployed	2 (2.7)	13 (12.4)	9 (9.4)	9 (8.7)

**Anthropometrics, mean (SD)**				
BMI(kg/m^2^),	30.7 (3.9)	31.0 (3.9)	30.7 (3.7)	31.0 (4.3)
Body fat (%)	47.8 (4.5)	47.6 (4.4)	47.9 (4.1)	48.0 (4.6)
Waist circumference (cm)	94.3 (11.3)	94.6 (10.2)	95.1 (10.1)	93.7 (9.9)

**Antidepressants/anxiolytics use, N (%)**				
Yes	29 (33.3)	35 (29.7)	41 (35.0)	44 (37.6)

**Lifestyle factors, mean (SD)**				
Aerobic fitness (ml/kg/min),	23.1 (4.1)	22.6 (3.8)	22.5 (4.1)	23.5 (4.1)
Physical activity (min/week)	23.8 (41.2)	33.6 (45.5)	37.7 (43.7)	33.6 (44.7)
Calorie intake (kcal/day) ^c^	1988 (669)	1884 (661)	1986 (589)	1890 (638)

**Psychosocial factors, mean (SD)**				
Depression	48.0 (9.0)	49.4 (9.8)	48.3 (9.4)	48.3 (8.7)
Anxiety	45.3 (7.0)	44.9 (6.8)	43.5 (6.1)	44.2 (6.8)
Perceived stress	3.71 (2.64)	3.47 (2.66)	3.43 (2.75)	3.04 (2.35)
Social support	81.0 (20.1)	80.0 (19.3)	81.4 (15.9)	81.7 (19.4)

**Health-related quality of life, mean (SD)**				
Physical functioning	86.8 (11.7)	86.2 (11.0)	87.8 (11.1)	86.7 (12.1)
Role-physical	81.6 (30.1)	83.5 (26.8)	82.8 (29.3)	83.5 (25.9)
Bodily pain	75.8 (17.2)	76.9 (15.1)	77.8 (16.5)	78.8 (16.8)
General health	57.1 (8.0)	55.9 (7.7)	56.9 (6.7)	57.6 (6.4)
Vitality	57.4 (16.0)	56.6 (17.7)	60.3 (16.3)	58.7 (18.6)
Social functioning	87.8 (18.0)	88.1 (17.1)	91.4 (13.1)	90.8 (13.4)
Role-emotional	84.1 (26.9)	82.2 (28.5)	87.5 (25.5)	88.6 (20.1)
Mental health	77.1 (13.5)	76.8 (13.1)	81.1 (10.0)	79.1 (12.3)

^a ^marital status (n = 438); ^b ^employment (n = 378); ^c ^calorie intake (n = 427)

### Intervention effects on weight, aerobic fitness and adherence

The intervention effects on weight and aerobic fitness and adherence were reported elsewhere [31]. In brief, the diet, exercise, and diet+exercise groups decreased body weight by 7.2 kg over 12 months (percent change from baseline body weight %Δ_Diet _= 8.5%; p < 0.01), 2.0 kg (%Δ_Exercise _= 2.4%, p = 0.03), and 8.9 kg (%Δ_Diet+Exercise _= 10.8%, p < 0.01), respectively compared with controls. Approximately half of the participants in the diet groups (diet 41.5%; diet + exercise groups 59.5%) achieved the goal of 10% weight reduction at 12 months. The exercise and diet + exercise groups met a mean 80% and 85% of the goal of 225 minutes per week of moderate intensity aerobic exercise, respectively. Aerobic fitness increased by 0.17 L/min and 0.12 L/min, respectively in exercise and diet+exercise groups (all p < 0.001, vs. control).

### Baseline HRQOL scores and psychosocial factors stratified by subgroups

Table 2 displays mean HRQOL scores at baseline stratified by baseline characteristics. Older women (≥ 57 years) had lower role-physical scores and perceived stress, and higher vitality scores compared to younger women (< 57 years; p < 0.05). None of the psychosocial factors and HRQOL scores were different between subgroups defined by ethnicity or education. Employed women had lower social functioning than unemployed women (p = 0.02). Women who were married or with partner reported higher levels of social support (p < 0.05; vs. no partner). Obese women had lower physical functioning and role-physical scores (p < 0.05; vs. overweight). Women taking antidepressants or anxiolytics reported a higher level of bodily pain; lower physical functioning, vitality, role-emotional, and mental health scores; and higher levels of depression and anxiety (all p < 0.05).

**Table 2:** Baseline scores of health-related quality of life (measured by SF-36) and psychosocial factors (depression and anxiety measured by BSI-18, perceived stress measured by the Perceived Stress Scale, social support measured by MOS Social Support Survey), stratified by subgroups

		Health-related quality of life (SF-36)								Psychosocial variables			
	N	PF	**RP**^c^	BP	GH	VT	SF	**RE**^d^	MH	DEP	ANX	PSS	SS
Demographics													
Age													
< 57 yrs	210	87.5	**86.7**†	76.7	57.2	**56.1**†	88.8	84.0	77.6	49.0	44.6	3.72*	79.2
≥ 57 yrs	228	86.3	**79.5**†	78.1	56.5	**60.3**†	90.4	87.3	79.6	48.1	44.2	3.09*	82.7
Ethnicity													
Non-Hispanic white	372	86.8	83.9	78.1	56.6	58.5	90.1	86.2	78.9	48.4	44.2	3.30	81.4
Others	66	87.4	77.7	73.9	58.2	57.2	87.1	83.1	76.9	49.6	45.4	3.89	78.9
Education													
No college degree	152	86.8	83.3	76.9	57.3	58.5	87.6	83.1	78.2	48.3	44.5	3.64	79.9
College degree	286	86.9	82.8	77.7	56.6	58.2	90.7	87.1	78.8	48.7	44.4	3.26	81.6
Employment **^a ^**													
Employed	344	87.2	83.4	77.0	56.7	57.7	88.7*	85.5	77.9	48.7	44.7	3.54	80.1
Unemployed	33	84.1	81.8	79.2	56.1	54.8	93.6*	85.9	79.6	47.7	44.1	2.91	83.8
Marital status **^b^**													
No partner	159	86.3	84.7	79.2	56.5	59.1	89.9	85.4	77.8	49.4	44.2	3.50	**72.4**†
Married or with partner	278	87.2	81.9	76.4	57.0	57.8	89.5	85.8	79.1	48.0	44.6	3.33	**86.0**†
Anthropometrics													
Overweight	209	**89.7**†	86.3*	79.0	56.8	59.7	90.7	86.7	78.6	48.0	44.2	3.19	82.1
Obese	229	**84.3**†	79.9*	76.1	56.9	57.0	88.6	84.8	78.7	49.0	44.6	3.58	80.0
Antidepressants/anxiolyticsuse													
No	289	**88.1**†	83.5	**79.2**†	57.3	**60.5**†	90.5	**88.4**†	**80.0**†	**47.5**†	**43.8**†	3.26	81.2
Yes	149	**84.5**†	81.9	**74.1**†	56.0	**54.1**†	88.0	**80.5**†	**76.1**†	**50.6**†	**45.6**†	3.66	80.6

*p < 0.05, †p < 0.01 comparing differences between subgroups

^a ^baseline employment (n = 377), **^b ^**marital status (n = 437), **^c ^**Role-physical (n = 437), **^d ^**Role-emotional (n = 436)

PF: physical functioning, RP: role-physical, BP: bodily pain, GH: general health, VT: vitality, SF: social functioning, RE: role-emotional, MH: mental health, DEP: depression, ANX: anxiety, PSS: perceived stress scale, SS: social support

### Intervention effects on 8 aspects of HRQOL

Overall, the 12-months changes in 4 subscales of HRQOL differed among the 4 groups: physical functioning (p < 0.001), role-physical (p < 0.001), vitality (p < 0.001), and mental health (p = 0.06) (Table 3). Compared with controls, the diet+exercise group increased physical functioning (p < 0.001), role-physical (p < 0.001), vitality (p < 0.001), and mental health scores (p = 0.01) and decreased bodily pain (p = 0.04). Although both the diet and diet+exercise groups increased vitality, the diet+exercise group showed a larger increase than the diet only group (p = 0.04 comparing the two groups). The diet only group increased vitality (p < 0.001; vs. controls) and mental health (p = 0.05; vs. controls). The exercise group did not improve any subscales of HRQOL compared with controls.

**Table 3:** Individual and combined effects of diet and/or exercise intervention on health-related quality of life scores (measured by SF-36)

	Baseline	12 months	Changes			
	**Unadjusted mean (SD)**	**Unadjusted mean (SD)**	**Unadjusted mean**	**Adjusted mean**	p-value *	**p-value ^†^**

**Physical functioning**						< 0.001
Control	86.8 (11.7)	84.5 (15.5)	-2.3	-2.6	Ref	
Diet	86.2 (11.0)	88.1 (15.9)	1.9	1.2	0.03	
Exercise	87.8 (11.1)	87.6 (15.0)	-0.2	-0.1	0.17	
Diet + Exercise	86.7 (12.1)	92.4 (11.3)	5.7	5.5	< 0.001 ^b^	

**Role-physical**						< 0.001
Control	81.6 (30.1)	78.7 (32.0)	-2.9	-3.7	Ref	
Diet	83.5 (26.8)	82.8 (30.4)	-0.7	-0.3	0.36	
Exercise	82.8 (29.3)	78.7 (32.7)	-4.1	-4.1	0.93	
Diet + Exercise	83.5 (25.9)	92.5 (18.9)	9.0	9.6	< 0.001 ^b^	

**Bodily pain**						0.12
Control	75.8 (17.2)	72.6 (18.2)	-3.2	-4.6	Ref	
Diet	76.9 (15.1)	76.8 (21.2)	-0.1	-1.1	0.15	
Exercise	77.8 (16.5)	74.5 (20.7)	-3.3	-3.8	0.74	
Diet + Exercise	78.8 (16.8)	79.1 (17.5)	0.3	0.4	0.04	

**General health**						0.57
Control	57.1 (8.0)	56.4 (7.1)	-0.7	-0.5	Ref	
Diet	55.9 (7.7)	56.9 (7.3)	1.0	0.5	0.24	
Exercise	56.9 (6.7)	56.4 (7.3)	-0.5	-0.5	0.97	
Diet + Exercise	57.6 (6.4)	56.9 (7.3)	-0.7	-0.3	0.81	

**Vitality**						< 0.001
Control	57.4 (16.0)	59.2 (17.9)	1.8	0.4	Ref	
Diet	56.6 (17.7)	65.7 (17.2)	9.1	7.2	< 0.001	
Exercise	60.3 (16.3)	62.9 (17.6)	2.6	2.8	0.25	
Diet + Exercise	58.7 (18.6)	70.2 (17.2)	11.5	11.2	< 0.001 ^a^	

**Social functioning**						0.43
Control	87.8 (18.0)	86.9 (17.5)	-0.9	-2.5	Ref	
Diet	88.1 (17.1)	87.2 (18.6)	-0.9	-3.1	0.83	
Exercise	91.4 (13.1)	88.5 (18.9)	-2.9	-4.0	0.58	
Diet + Exercise	90.8 (13.4)	91.6 (17.0)	0.8	-0.2	0.37	

**Role-emotional**						0.09
Control	84.1 (26.9)	83.3 (31.8)	-0.8	-3.3	Ref	
Diet	82.2 (28.5)	85.6 (27.4)	3.4	-0.8	0.51	
Exercise	87.5 (25.5)	81.4 (32.5)	-6.1	-6.2	0.45	
Diet + Exercise	88.6 (20.1)	90.3 (22.8)	1.7	2.5	0.13	

**Mental health**						0.06
Control	77.1 (13.5)	77.3 (14.5)	0.2	-0.8	Ref	
Diet	76.8 (13.1)	80.2 (13.0)	3.4	2.2	0.05	
Exercise	81.1 (10.0)	81.2 (11.7)	0.1	0.9	0.29	
Diet + Exercise	79.1 (12.3)	82.3 (12.6)	3.2	3.1	0.01	

Adjusted mean change indicates adjustment for the baseline health-related quality of life (HRQOL) scores and covariates

*p-value comparing 12-month changes in HRQOL vs. control adjusting for the baseline scores and covariates (Physical functioning: baseline BMI, medication use, Role-physical: age, baseline BMI, Bodily pain: medication use, Vitality: age, medication use, Social functioning: employment status, Role-emotional: medication use, Mental health: medication use)

^†^p-value for group effects on 12-month changes in HRQOL adjusting for baseline scores and covariates (Physical functioning: baseline BMI, medication use, Role-physical: age, baseline BMI, Bodily pain: medication use, Vitality: age, medication use, Social functioning: employment status, Role-emotional: medication use, Mental health: medication use)

^a^p-value< 0.05 vs. diet group, ^b^p-value< 0.01 vs. diet group

### Intervention effects on psychosocial variables

The 12-month change in perceived stress differed by study arm (p = 0.04). The diet+exercise group significantly decreased perceived stress (-0.55 points) while the control group increased their stress levels (0.32 points) (p = 0.006) (Table 4). Although the overall and pairwise comparisons among 4 study arms did not reach statistical significance (due to the Bonferroni correction for multiple comparison; p ≤0.017 was considered statistically significant in the pairwise comparision), the diet+exercise group reduced depression (Δ_Diet+Exercise _= -1.7 points, p = 0.03; vs. control Δ_Control _= 0.7 points) and increased social support (Δ_Diet+Exercise _= 1.0 points, p = 0.05; vs. control Δ_Control_= -2.8 points).

**Table 4:** Individual and combined effects of diet and/or exercise intervention on psychosocial factors (depression and anxiety measured by BSI-18, perceived stress measured by the Perceived Stress Scale, social support measured by MOS Social Support Survey)

	Baseline	12 months	Changes			
	**Unadjusted mean (SD)**	**Unadjusted mean (SD)**	**Unadjusted mean**	**Adjusted mean**	p-value *	**p-value ^†^**

**Depression**						0.12
Control	48.0 (9.0)	48.4 (9.6)	0.4	0.7	Ref	
Diet	49.4 (9.8)	47.8 (8.7)	-1.6	-0.5	0.31	
Exercise	48.3 (9.4)	48.1 (9.8)	-0.2	0.2	0.68	
Diet + Exercise	48.3 (8.7)	46.2 (8.2)	-2.1	-1.7	0.03	

**Anxiety**						0.41
Control	45.3 (7.0)	45.3 (8.7)	0.0	0.6	Ref	
Diet	44.9 (6.8)	43.8 (7.3)	-1.1	-0.6	0.17	
Exercise	43.5 (6.1)	43.0 (6.9)	-0.5	-0.7	0.14	
Diet + Exercise	44.2 (6.8)	43.5 (6.4)	-0.7	-0.6	0.15	

**Perceived stress**						0.04
Control	3.71 (2.64)	3.89 (2.75)	0.18	0.32	Ref	
Diet	3.47 (2.66)	3.51 (2.65)	0.04	0.08	0.44	
Exercise	3.43 (2.75)	3.35 (2.84)	-0.08	-0.06	0.23	
Diet + Exercise	3.04 (2.35)	2.66 (2.27)	-0.38	-0.55	0.006	

**Social support**						0.11
Control	81.0 (20.1)	78.5 (20.8)	-2.5	-2.8	Ref	
Diet	80.0 (19.3)	79.4 (20.5)	-0.6	-1.1	0.38	
Exercise	81.4 (15.9)	78.6 (20.8)	-2.8	-2.9	0.97	
Diet + Exercise	81.7 (19.4)	82.9 (18.6)	1.2	1.0	0.05	

Adjusted means are changes in psychological factors adjusted for baseline scores and covariates (e.g., age, baseline BMI, marital status, anxiolytics and antidepressants use)

*p-value comparing 12-month changes in psychosocial factors vs. control adjusting for the baseline scores and covariates (Depression: medication use, Anxiety: medication use, Stress: age, Social support: marital status)

^†^p-value for group effects on 12-month changes in psychosocial factors adjusting for baseline scores and covariates (Depression: medication use, Anxiety: medication use, Stress: age, Social support: marital status)

### Bivariate correlations between changes in HRQOL and physical and psychosocial factors

Bivariate correlations were examined for 12-month changes in HRQOL and factors that significantly changed during the intervention using combined data of all 4 study groups (Table 5). Weight loss was positively associated with changes in physical functioning (r = 0.28, p < 0.001), role-physical (r = 0.18, p < 0.001), vitality (r = 0.36, p < 0.001) and mental health scores (r = 0.13, p = 0.006). Weight loss was also associated with an improvement in depression scores (r = -0.11, p = 0.02). Increased aerobic fitness was positively associated with physical functioning scores (r = 0.16, p = 0.0007). Decreased depression and perceived stress, and improved social support were associated with increases in physical functioning, role-physical, vitality and mental health scores (all p < 0.001). Decreased depression was associated with increased physical functioning (r = -0.21, p < 0.001), role-physical (r = -0.23, p < 0.001), vitality (r = -0.42, p < 0.001), and mental health scores (r = -0.55, p < 0.001). Increased stress was inversely associated with physical functioning (r = -0.22, p < 0.001), role-physical (r = -0.20, p < 0.001), vitality (r = -0.32, p < 0.001), and mental health scores (r = -0.51, p < 0.001). Increased social support was associated with improved physical functioning (r = 0.24, p < 0.001), role-physical (r = 0.22, p < 0.001), vitality (r = 0.22, p < 0.001), and mental health (r = 0.25, p < 0.001).

**Table 5:** Bivariate correlations between 12-month changes in health-related quality of life (measured by SF-36) and potential predictors

	Δ Weight		Δ Aerobic fitness		Δ Depression		Δ Perceived stress		Δ Social support	
	
	R	p	r	p	R	p	r	p	r	p
Δ Weight	---	---	**-**0.02	0.64	**0.11**	**0.02**	0.07	0.17	-0.02	0.66
Δ Aerobic fitness	-0.02	0.64	---	---	-0.0006	0.99	-0.08	0.08	0.02	0.61
Δ Physical functioning	**-0.28**	**< 0.001**	**0.16**	**< 0.001**	**-0.21**	**< 0.001**	**-0.22**	**< 0.001**	**0.24**	**< 0.001**
Δ Role-physical	**-0.18**	**< 0.001**	0.05	0.26	**-0.23**	**< 0.001**	**-0.20**	**< 0.001**	**0.22**	**< 0.001**
Δ Vitality	**-0.36**	**< 0.001**	0.06	0.22	**-0.42**	**< 0.001**	**-0.32**	**< 0.001**	**0.22**	**< 0.001**
Δ Mental health	**-0.13**	**0.006**	0.04	0.43	**-0.55**	**< 0.001**	**-0.51**	**< 0.001**	**0.25**	**< 0.001**

### Predictors of 12-month changes in HRQOL

The 12-month changes in the four subscales of HRQOL that significantly differed by intervention arm (physical functioning, role-physical, vitality, and mental health) were further examined to identify the predictors of HRQOL change (Table 6). Change in anxiety levels did not differ by intervention arm; therefore, it was not included in the model [32]. In multiple regression models, the 12-month changes in weight (β = -0.50, p < 0.001), aerobic fitness (β = 4.67, p = 0.01), perceived stress (β = -0.58, p = 0.02), and social support (β = 0.17, p < 0.001) predicted increased physical functioning. Reduced weight (β = -0.67, p = 0.001) and depression (β = -0.50, p = 0.001) and improved social support (β = 0.24, p = 0.01) predicted increased role-physical score. Reduced weight (β = -0.74, p < 0.001), depression (β = -0.42, p < 0.001) and perceived stress (β = -0.79, p = 0.004) were associated with improved vitality. Weight loss (β = -0.15, p = 0.04) and decreases in depression (β = -0.43, p < 0.001) and perceived stress (β = -1.28, p < 0.001) predicted positive changes in mental health.

**Table 6:** Predictors of 12-month changes in health-related quality of life (measured by SF-36)

	12-month changes in HRQOL							
	**Physical functioning**		**Role-physical**		**Vitality**		**Mental health**	

	**β**	**P**	**β**	**P**	**β**	**P**	**β**	**P**
Change in weight	**-0.50**	**< 0.001**	**-0.67**	**0.001**	**-0.74**	**< 0.001**	**-0.15**	**0.04**
Change in aerobic fitness	**4.67**	**0.01**	3.65	0.37	0.93	0.65	-0.15	0.91

Change in depression	-0.12	0.10	**-0.50**	**0.001**	**-0.42**	**< 0.001**	**-0.43**	**< 0.001**
Change in perceived stress	**-0.58**	**0.02**	-0.66	0.24	**-0.79**	**0.004**	**-1.28**	**< 0.001**
Change in social support	**0.17**	**< 0.001**	**0.24**	**0.01**	0.08	0.07	0.04	0.18

The regression models were adjusted for group assignment, baseline health-related quality of life (HRQOL) scores, and covariates (Physical functioning: baseline BMI, medication use, Role-physical: age, baseline BMI, Vitality: age, medication use, Mental health: medication use)

We also performed the analyses using available data and using multiple imputation. There were no substantial differences between the results on these analyses except for the relationship between changes in aerobic fitness and the physical functioning scale. The correlation coefficient between 12-month changes in aerobic fitness and the physical functioning scale was significant in the last-observation carried forward and complete case analyses (p < 0.01), while it was non-significant in the multiple imputation analyses (p = 0.09, data are available on request). Therefore, we presented the results of last observation carried forward analyses in this paper. The analysis results did not differ substantially when the covariates were removed from the model (unadjusted model, supplementary tables are available on request).

## Discussion

This study examined the individual and combined effects of dietary weight loss and/or aerobic exercise interventions on HRQOL among sedentary, overweight/obese postmenopausal women. To our knowledge, this trial is the first to compare individual and combined effects of dietary weight loss and exercise intervention on HRQOL in overweight/obese, postmenopausal women without major medical conditions. We found that the combined dietary weight loss and exercise group improved more aspects of HRQOL and psychosocial factors (depression, stress and social support) with larger increments compared with diet or exercise alone. We also found significant associations between weight loss, increased aerobic fitness, and improvements in HRQOL and psychological factors, suggesting that these factors may explain, at least in part, the improved HRQOL observed in the diet and exercise interventions.

The combined dietary weight loss and exercise group improved more aspects of HRQOL and with larger increments compared with diet or exercise alone. Our findings were consistent with previous trials in clinical populations, among those with type 2 diabetes [13] or osteoarthritis [14]. The latter trial reported up to a 16.5 point increase in all subscales of SF-36 with a 18-month diet+exercise intervention [14], which was greater than the observed changes in our sample (5-11 points). This may be caused by differences in the study sample, as the observed increase in HRQOL scores among our combined diet+exercise group was consistent with previous weight loss trials in general populations [4,17]. In a 6-month weight loss trial (low calorie diet and aerobic exercise) among 298 obese women (age 50-75), women lost 9.4% of baseline weight and increased physical functioning and vitality scores by 6 and 8 points, respectively [17]. Another 6-month weight loss trial in 144 overweight/obese adults reported a mean weight loss of 5.6 kg and 2 to 11-point improvements in 8 subscales of SF-36 [4].

In contrast to a number of studies reporting positive effects of exercise on HRQOL, we did not find significant improvements in any aspects of HRQOL in women randomized to the exercise-only group. It is possible that our participants had high baseline HRQOL which could have caused a ceiling effect. Preference for type of exercise could also have affected the results. Courneya et al. found that participants who preferred resistant training showed greater increase in HRQOL when assigned to resistant training group compared with those assigned to aerobic exercise or control groups [33]. Our participants might have preferred to be assigned to a group other than the exercise-only group, which could have resulted in minimal changes in HRQOL.

The combined diet+exercise intervention also improved psychosocial factors (depression, stress, and social support), while there were no effects on these factors in the diet or exercise alone groups. Although we are not aware of studies comparing these psychological outcomes in individual vs. combined diet and exercise interventions, lifestyle modification programs involving diet and exercise have been shown to improve psychological health. A 12-month intensive lifestyle intervention program of the Look AHEAD (Action for Health in Diabetes) Trial, mediated through weight loss (mean 8.8 kg weight loss among intervention group) and aerobic fitness, improved depression in 4223 overweight adults with type 2 diabetes [18]. A cardiac rehabilitation program reduced stress, which was associated with weight loss and improved aerobic fitness [34]. Our finding that the combined diet+exercise group improved psychological factors is consistent with these studies, but the reasons for the improvements are not clear. We did not find any significant correlations between weight loss or aerobic fitness with these psychosocial factors except for a correlation between weight loss and reduced depression. Future studies are recommended to investigate mechanisms by which lifestyle interventions may improve psychological health.

Positive changes in depression and stress were significantly associated with 4 subscales of HRQOL, which remained significant after adjusting for changes in weight and aerobic fitness. Studies have shown that psychological disorders affect various aspects of HRQOL. An analysis of 11,242 outpatients in the U.S. showed that individuals who are depressed have lower physical functioning, role-physical and social functioning compared with non-depressed individuals [35]. Another study has shown that increased depressive symptoms were associated with decline in all 8 aspects of SF-36 among female patients with remitted major depression disorder [36]. Our study confirmed that psychological conditions have a significant impact on HRQOL and that a lifestyle behavioral change of a diet and exercise in combination, is a potential method to improve psychological health.

Improved aerobic fitness was an independent predictor of 12-month changes in physical functioning. Consistent with our findings, Ross et al. found that changes in BMI and aerobic fitness independently explained a change in physical functioning score, and that improved aerobic fitness had independent effects beyond BMI change only in physical functioning scale among 8 subscales of SF-36 in a 6-month lifestyle intervention among obese women [17]. An analysis from the Look AHEAD trial found that both weight loss and increased aerobic fitness mediated the intervention effects on physical composite scores [18]. In our previous 12-month exercise trial in 173 postmenopausal women, we found that a change in aerobic fitness was associated with a change in physical functioning but not with changes in either mental health or general health [6].

Weight loss in the present study was associated with improvements in both physical and mental aspects of HRQOL. A 12-month follow-up of a 6-month lifestyle intervention found that individuals who continued to lose weight during the follow-up period showed improved vitality and general health of SF-36 and that weight loss was associated with improvements in these aspects of SF-36 among 508 postmenopausal women [37]. Our findings confirmed that obesity is a risk factor for reduced HRQOL and that weight loss can improve both physical and mental aspects of HRQOL.

Previous studies have shown an important role of psychosocial factors on explaining how exercise impacts quality of life [38-41]. In multiple sclerosis patients, depression, social support, self-efficacy and fatigue mediated effects of exercise on quality of life [41]. Greater social support was associated with stronger exercise self-efficacy in older adults in another study [42]. Exercise self-efficacy mediated the exercise effect on mental and physical aspects of HRQOL in older women [40]. Higher exercise self-efficacy was associated with greater physical power score, a combined score of aerobic fitness and five items from the Senior Fitness Test [43] among older adults [44]. It is possible that the observed associations of weight loss and improved aerobic fitness with HRQOL in our study could be mediated through increase in exercise self-efficacy. Future studies may benefit from testing psychosocial predictors of quality of life including self-efficacy to further determine the mechanism of how interventions affect HRQOL.

The strengths of this trial include its large sample size; randomized controlled design; three intervention arms allowing direct comparisons of individual and combined exercise and diet groups to each other and controls; excellent adherence to intervention prescription; low rate of drop-outs (9%); and use of validated measures of HRQOL and psychosocial factors. In particular, direct comparison between combined diet+exercise and diet or exercise alone allowed us to understand the individual and combined contribution of these lifestyle behaviors on HRQOL.

This study is limited by some factors that should be kept in mind when interpreting the results. Our sample consisted primarily of non-Hispanic White women with a high education level on average. Hence, our findings may not be generalizable to men, or women in other ethnic groups or with different education levels. Another limitation is the relatively high HRQOL scores among our sample. Even though we found significant effects on several aspects of HRQOL, the analysis may have suffered from a ceiling effect. Based on these limitations, future studies are needed to test the effects of these dietary weight loss and exercise interventions in other populations such as women of other race/ethnicity groups or in men.

## Conclusions

Our findings suggest that the combination of dietary weight loss and exercise may have a larger beneficial effect on HRQOL compared with dietary weight loss or exercise alone. Weight loss and improvements in aerobic fitness and psychosocial factors (depression, stress, and social support) were predictors of increased HRQOL, suggesting that these factors could mediate the intervention effects on HRQOL.

## Abbreviations

ANCOVA: analysis of covariance; ANOVA: analysis of variance; BMI: body mass index; BSI: Brief Symptom Inventory; DPP: Diabetes Prevention Program; HRQOL: health related quality of life; Look AHEAD: Action for health in Diabetes; MOS: Medical Outcome Study Social Support Survey; SF-36: Medical Outcomes Study 36-Item Short-Form Health Survey.

## Competing interests

The authors declare that they have no competing interests.

## Authors' contributions

II conducted data analyses, interpreted the results and drafted the manuscript. CMA interpreted the results and drafted the manuscript. AK and CEB acquired the data. LX performed analysis. GLB designed the study. AM designed the study, acquired the data, interpreted the results, and drafted the manuscript. All authors have revised and approved the manuscript.
